# Kynurenic Acid Protects Against Ischemia/Reperfusion-Induced Retinal Ganglion Cell Death in Mice

**DOI:** 10.3390/ijms21051795

**Published:** 2020-03-05

**Authors:** Rooban B. Nahomi, Mi-Hyun Nam, Johanna Rankenberg, Stefan Rakete, Julie A. Houck, Ginger C. Johnson, Dorota L. Stankowska, Mina B. Pantcheva, Paul S. MacLean, Ram H. Nagaraj

**Affiliations:** 1 Sue Anschutz-Rodgers Eye Center and Department of Ophthalmology, University of Colorado, Aurora, CO 80045, USA; mi-hyun.nam@cuanschutz.edu (M.-H.N.); johanna.rankenberg@cuanschutz.edu (J.R.); stefan.rakete@med.uni-muenchen.de (S.R.); mina.pantcheva@cuanschutz.edu (M.B.P.); 2 Division of Endocrinology, Metabolism and Diabetes, School of Medicine, University of Colorado, Aurora, CO 80045, USA; julie.houck@cuanschutz.edu (J.A.H.); ginger.johnson@cuanschutz.edu (G.C.J.); paul.maclean@cuanschutz.edu (P.S.M.); 3 Department of Pharmacology and Neuroscience, North Texas Eye Research Institute, University of North Texas Health Science Center, Fort Worth, TX 76107, USA; dorota.stankowska@unthsc.edu; 4 Skaggs School of Pharmacy and Pharmaceutical Sciences, University of Colorado, Aurora, CO 80045, USA

**Keywords:** kynurenic acid, kynurenine 3-monooxygenase, ischemia/reperfusion, diabetes, retinal ganglion cells, neuroprotection

## Abstract

Background: Glaucoma is an optic neuropathy and involves the progressive degeneration of retinal ganglion cells (RGCs), which leads to blindness in patients. We investigated the role of the neuroprotective kynurenic acid (KYNA) in RGC death against retinal ischemia/reperfusion (I/R) injury. Methods: We injected KYNA intravenously or intravitreally to mice. We generated a knockout mouse strain of kynurenine 3-monooxygenase (KMO), an enzyme in the kynurenine pathway that produces neurotoxic 3-hydroxykynurenine. To test the effect of mild hyperglycemia on RGC protection, we used streptozotocin (STZ) induced diabetic mice. Retinal I/R injury was induced by increasing intraocular pressure for 60 min followed by reperfusion and RGC numbers were counted in the retinal flat mounts. Results: Intravenous or intravitreal administration of KYNA protected RGCs against I/R injury. The I/R injury caused a greater loss of RGCs in wild type than in KMO knockout mice. KMO knockout mice had mildly higher levels of fasting blood glucose than wild type mice. Diabetic mice showed significantly lower loss of RGCs when compared with non-diabetic mice subjected to I/R injury. Conclusion: Together, our study suggests that the absence of KMO protects RGCs against I/R injury, through mechanisms that likely involve higher levels of KYNA and glucose.

## 1. Introduction

Glaucoma affects nearly 60 million people worldwide and approximately 8 million people are blind from this disease [1]. In the US, more than 2.9 million people are afflicted with glaucoma [2]. Axonal degeneration and the subsequent death of retinal ganglion cells (RGCs) are the primary reasons for vision loss in glaucoma [3,4,5]. RGC death can occur from excitotoxic damage, neurotrophic factor deprivation, oxidative stress, inflammation, mitochondrial dysfunction and axonal transport failure [3,6]. The fact that several mechanisms are involved in the pathogenesis of glaucoma suggests that multiple factors collectively cause RGC death. The interplay between these collective factors, and the exact mechanisms leading to RGC death are not fully understood. RGC death is reproducible in several animal models, and therapies aimed at preventing RGC death have shown success in animals [7]. However, further work is necessary for a deeper understanding of the biochemical mechanisms and development of innovative therapies against RGC death.

**Figure 1:**
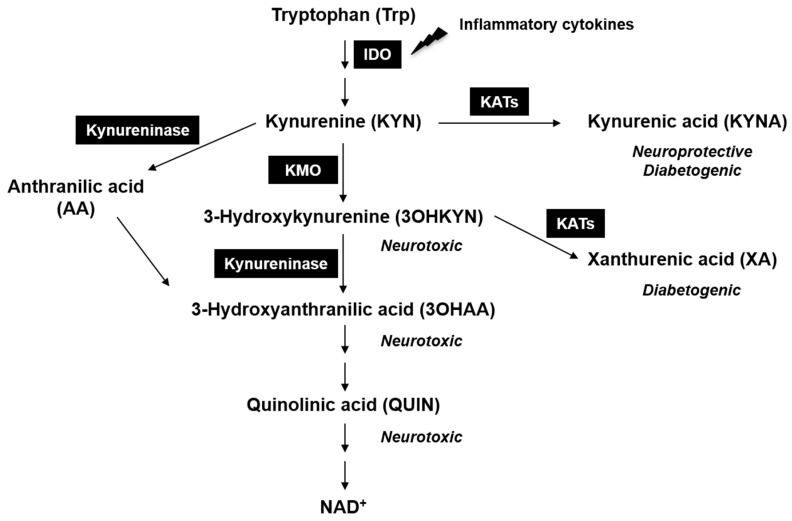
The kynurenine pathway (KP) and its metabolites. Indoleamine 2,3-dioxygenase (IDO) is the first enzyme in the KP. IDO synthesis is stimulated by inflammatory cytokines. Kynurenine 3-monooxygenase (KMO) converts kynurenine (KYN) to neurotoxic 3-hydroxykynurenine (3OHKYN). Quinolinic acid (QUIN), which is an N-methyl-D-aspartate (NMDA) receptor agonist, is also formed in this pathway. Kynurenine aminotransferases (KATs) convert KYN to neuroprotective kynurenic acid (KYNA) and kynureninase convert KYN to anthranilic acid (AA).

The breakdown of tryptophan (Trp) via the kynurenine pathway (KP) (Figure 1) is initiated by indoleamine 2,3-dioxygenase (IDO). IDO synthesis is stimulated by interferon gamma and other pro-inflammatory cytokines [8,9,10]. It has been reported that pro-inflammatory cytokines are elevated in the eyes of animals during experimental models of glaucoma [11,12,13,14]. IDO catalyzes the conversion of Trp to N-formyl kynurenine, which is then converted to kynurenine (KYN) by kynurenine formamidase. KYN can be converted to kynurenic acid (KYNA) by kynurenine amino transferases (KATs). KYNA is considered as neuroprotective because it is an antagonist for the N-methyl-D-aspartic acid (NMDA) receptor [15].

Kynurenine 3-monooxygenase (KMO) is a flavin adenine dinucleotide-dependent enzyme, localized to the outer mitochondrial membrane. It is predominantly expressed in the brain, liver and kidney [16,17]. The absence of KMO leads to the accumulation of its substrate KYN, which in turn results in increased anthranilic acid (AA) and KYNA levels [18]. 3-Hydroxykynurenine (3OHKYN) is derived from KYN by an enzymatic mechanism catalyzed by KMO. 3OHKYN can be further enzymatically converted to 3-hydroxy anthranilic acid (3OHAA, neurotoxic) quinolic acid (QUIN), an agonist of the NMDA receptor that enhances excitotoxicity [19]. The terminal product in the KP is oxidized nicotinamide adenine dinucleotide (NAD^+^). The KP is also implicated in various neurological diseases [20]. High levels of QUIN and 3OHKYN and reduced levels of KYNA have been observed in the brains of Huntington’s disease patients [21,22,23]. It has been found that traumatic brain injury activates the KP resulting in elevated QUIN [24], and another study showed that KMO plays an important role in RGC death in mice subjected to traumatic brain injury [25]. Previous studies have shown that the absence of KMO activity resulted in beneficial effects by reducing inflammation and a reduction in peripheral lipopolysaccharide-induced depressive-like behaviors in mice [26]. Furthermore, in rodent disease models of acute pancreatitis, the absence of active KMO significantly protected internal organs from pathological changes [27]. A recent study showed that the absence of KMO protected kidneys from ischemia/reperfusion (I/R) injury in mice [28]. 

The objective of this study was to determine the role of KYNA in RGC death in an acute I/R injury model in mice. To accomplish this, we administered KYNA as well as developed a mouse KMO knockout (KO) strain. We report below that the absence of KMO offers protection against RGC loss induced by I/R injury. Interestingly, during the course of this study, we noticed that the KMO KO mice had mildly elevated fasting blood glucose (FBG) levels. This finding led us to undertake further studies focused on glucose metabolism. We found that the insulin tolerance test (ITT) and body weights were comparable between the wild type (WT) and KMO KO mice.

## 2. Results

### 2.1. Administration of KYNA Inhibited I/R Injury-Mediated RGC Loss in WT Mice

To determine whether the KYNA is neuroprotective in the retina, we first investigated whether it is permeable to the blood retinal barrier, although KYNA has been reported to be poorly permeable to the blood brain barrier [29,30]. We injected KYNA intravenously into WT mice and measured its levels in the serum and retina after 2 h. We found a significant increase in KYNA levels both in serum and retinas of KYNA injected mice compared to sodium phosphate buffer-injected control mice (Supplementary Figure S1). A similar trend was observed in the retinas of mice subjected to I/R injury (prior to KYNA injection) (Supplementary Figure S1).

**Figure 2:**
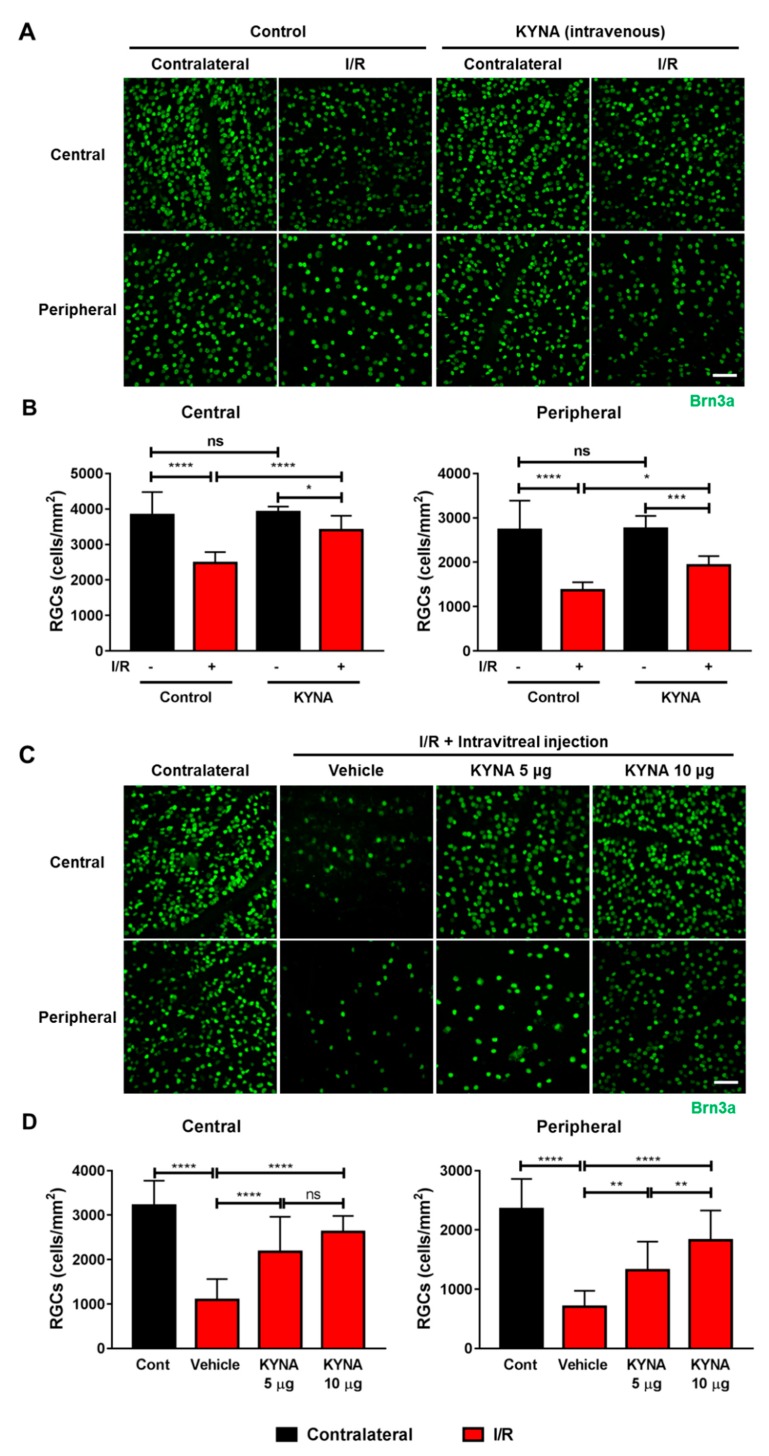
Retinal ganglion cell (RGC) loss in WT mice was blocked by intravenously and intravitreally injected KYNA. I/R injury was performed as described in the methods. Mice were intravenously injected with either vehicle alone or KYNA immediately after and 24 h after I/R injury (*n* = 5). Retinas were dissected out 14 days after I/R injury, flat mounted and immunostained for Brn3a (**A**,**B**) (*n* = 5). KYNA (5 μg (*n* = 8) or 10 μg (*n* = 7)) or vehicle alone was intravitreally injected immediately after I/R injury, and after 24 h. Retinas were dissected out after 14 days after I/R injury, flat mounted and immunostained for Brn3a (**C**,**D**) (*n* = 7–8), ns = not significant, * *p* < 0.05, ** *p* < 0.01, *** *p* < 0.001, and **** *p* < 0.0001. Scale bar = 50 μm.

WT mice subjected to I/R injury and given KYNA intravenously (immediately after, and 24 h after I/R injury) showed reduced RGC loss (brain-specific homeobox/POU domain protein 3A (Brn3a)-positive); the loss was 36.3% and 50% in the central and peripheral retina of I/R group, and it was 11.2% and 29.4% in the I/R + KYNA group (Figure 2A,B). The protective effect appeared to be better in the central retina than the peripheral retina. We also found similar RGC protective effects of KYNA in mice subjected to I/R injury by the intravitreally administered KYNA (Figure 2C,D). KYNA was administered at two doses (5 and 10 μg). The RGC loss was 32.4% and 46.1% in the central and peripheral retina of I/R-injured + 5 μg KYNA, when compared to 66.1% and 70.9% in the I/R-injured retinas. The RGC loss was further reduced in I/R-injured + 10 μg KYNA (18.5% and 26.8% in the central and peripheral retina).

### 2.2. The Absence of KMO Resulted in Altered Levels of KP Metabolites in the Retina

**Figure 3:**
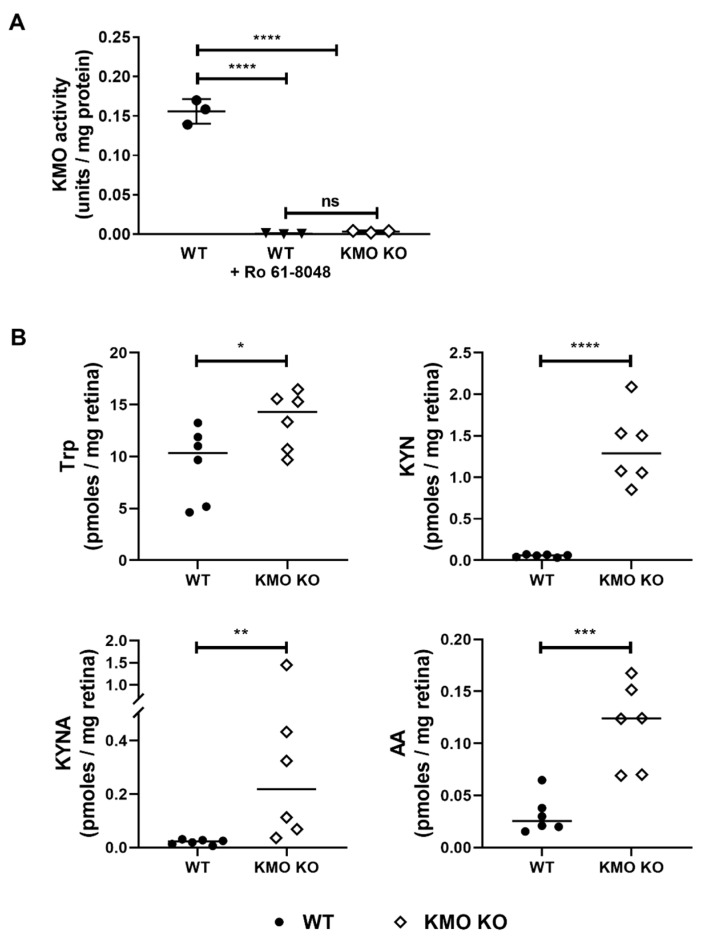
KMO activity was present in wild type (WT) but not in KMO knockout (KO) mice, and serum KP metabolites were altered in KMO KO mice. KMO activity was measured in livers isolated from WT and KMO KO mice (**A**). The KMO activity in WT liver isolate was blocked by a KMO inhibitor Ro61-8048, confirming that the observed activity was due KMO (*n* = 3). Retinal KP metabolites were higher in the KMO KO than in WT mice **(B)**. WT and KMO KO mice (*n* = 6) were euthanized and KP metabolites were determined by LC-MS/MS. ns = not significant, * *p* < 0.05, ** *p* < 0.01, *** *p* < 0.001, and **** *p* < 0.0001.

Next, we generated KMO KO mouse to elevate KYNA levels de novo in the retina. After generation of the KO mice without the presence of the Rd8 point mutation (determined by genotyping the *Kmo^-/-^ Rd8^WT/WT^* mice, qPCR and RT-PCR in retinas (Supplementary Figure S2), we measured the KMO activity in the liver. Significant KMO enzyme activity was observed in the WT mice (Figure 3A), which could be inhibited by the addition of Ro61-8048, whereas samples from the KO mice did not show this activity, confirming the absence of KMO. Next, we checked the KP metabolites in the serum and retinas from these animals by LC-MS/MS. The serum levels of the KP metabolites KYN, KYNA and AA were higher in the KMO KO than WT mice (Supplementary Figure S3). We also found significantly higher levels of Trp, KYN, KYNA, and AA in the retinas of KMO KO mice compared to WT mice (Figure 3B). The morphology of the retinas from WT and KMO KO mice was similar, as observed by H&E staining (Supplementary Figure S4). KMO KO mice had normal retinal vasculature compared to age-matched WT animals (Supplementary Figure S5).

### 2.3. The Absence of KMO Inhibited I/R Injury-Mediated Loss of Brn3a-Positive RGCs

**Figure 4:**
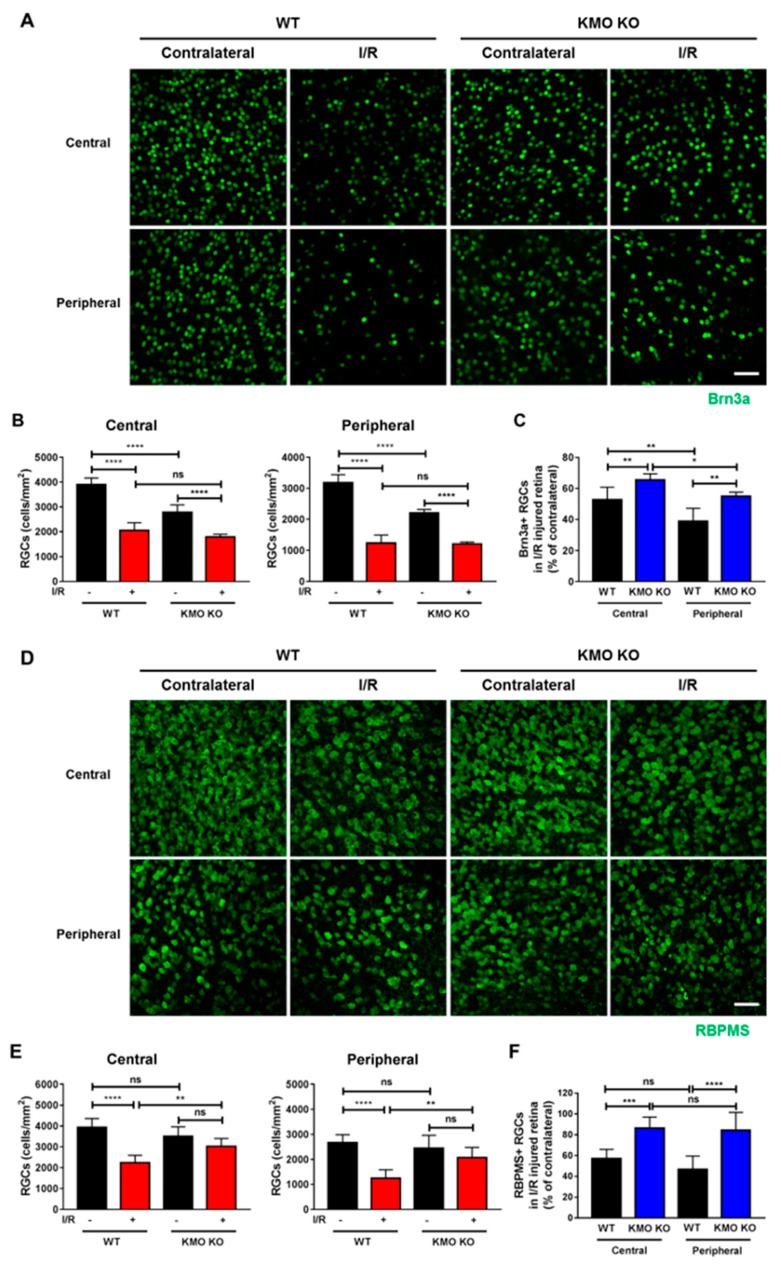
RGCs in KMO KO mice were protected from I/R injury. Retinas were dissected out 14 days after I/R injury, flat mounted and immunostained for Brn3a (**A**–**C**) (*n* = 3) or RBPMS (**D**–**F**) (WT; *n* = 4, KMO KO; *n* = 5). I/R injury reduced RGC numbers (central and peripheral retinas) in both WT and KMO KO mice. The percentage of Brn3a-positive and RBPMS-positive RGCs following I/R injury was higher in KMO KO mice compared to WT mice in both the central and peripheral retinas (**C,F**). The bar graphs represent the means ± SD of triplicate measurements. ns = not significant, * *p* < 0.05, ** *p* < 0.01, *** *p* < 0.001, and **** *p* < 0.0001. Scale bar = 50 μm. RBPMS = RNA-binding protein with multiple splicing.

To determine whether RGCs are protected from I/R-induced damage in mice lacking KMO, WT and KMO KO mice were subjected to I/R injury. I/R injury significantly decreased the Brn3a-positive RGC numbers both in the central and peripheral retinas from both WT and KMO KO mice (Figure 4A,B). However, the percentage of Brn3a-positive RGCs was significantly higher in both the central and peripheral retinas of KMO KO mice relative to WT mice following I/R (Figure 4C). Since there was no difference in the number of remaining RGCs after I/R between WT and KMO KO mice, we carried out dual staining of Brn3a and RNA-binding protein with multiple splicing (RBPMS) in WT and KMO KO mouse retinas. The RGC subpopulations were different between the KMO KO and WT mice, as indicated by a significantly lower numbers of Brn3a-positive RGCs in the KMO KO mice, and marginally (statistically insignificant) lower numbers in RBPMS-positive RGCs compared to the WT mice (Supplementary Figure S6). Further, we found a significant reduction in RBPMS-positive RGCs in WT mice subjected to I/R injury, which was less severe in the KMO KO mice (Figure 4D,E). In I/R injured retinas the percentage of RBPMS-positive RGCs was significantly higher in both the central and peripheral retinas of KMO KO mice relative to WT mice (Figure 4F).

### 2.4. The Absence of KMO Led to Higher FBG but No Significant Differences in Body Weight, Glucose Tolerance, or Insulin Insensitivity

**Figure 5:**
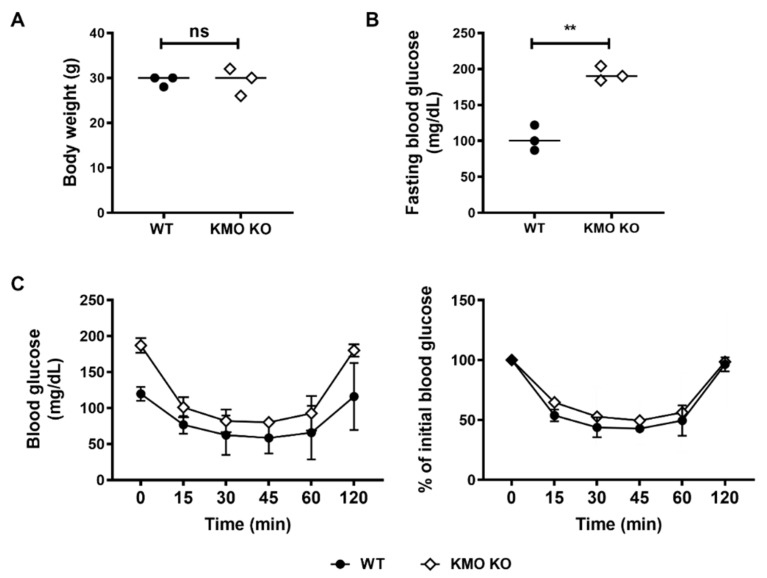
Fasting blood glucose was higher in KMO KO mice than in WT mice, but body weights and insulin tolerance test results were similar. The body weights were similar in WT and KMO KO mice (**A**). Fasting blood glucose (FBG) was higher in KMO KO mice (**B**). The KMO KO mice responded to insulin like the WT mice (**C**). The line graphs represent the means ± SD of triplicate measurements. ns = not significant, ** *p* < 0.01.

A previous study has shown that xanthurenic acid (XA) and KYNA inhibit pro-insulin synthesis in isolated rat pancreatic islets [31]. The possible mechanisms of the diabetogenic effect of KYNA might be related to the NMDA antagonist function that can inactivate the inhibition of glucose production induced by NMDA agonists [32]. We have observed that the body weights were not significantly different in between the WT and KMO KO mice (Figure 5A). However, KMO KO mice showed higher FBG levels than WT mice (Figure 5B). To pursue the underlying mechanism of the higher FBG levels, we assessed insulin sensitivity via a standard ITT. Mice were intraperitoneally (i.p.) administered insulin and blood glucose was monitored for 120 min. As shown in Figure 5C, KMO KO mice and WT mice exhibited a similar response, as indicated by the comparable reduction in initial blood glucose levels and the percent drop from the initial levels (Figure 5C). Furthermore, there were no differences in serum insulin between WT and KMO KO as observed in ELISA (Supplementary Figure S7).

### 2.5. Elevated Blood Glucose Protected RGC in WT Mice Subjected to I/R Injury

**Figure 6:**
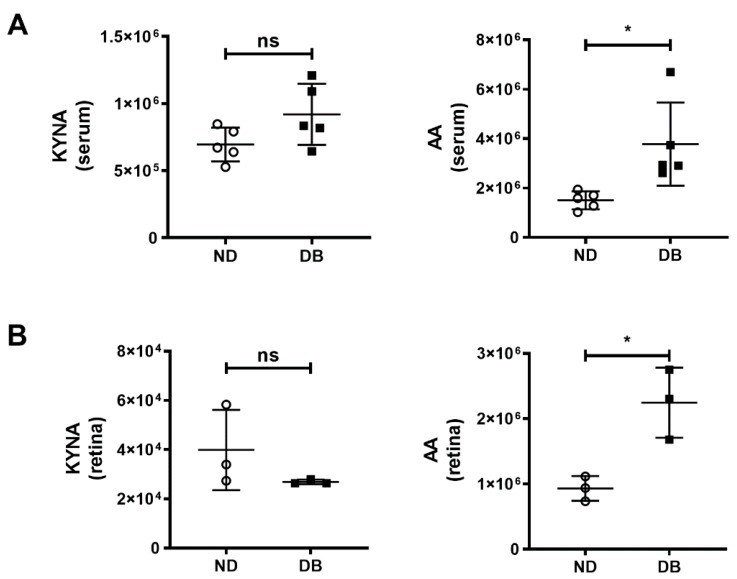
The KYNA and AA were measured by LC-MS/MS (area under curve) in (**A**) serum (*n* = 5) and (**B**) retinas (*n* = 3) of non-diabetic (ND) and diabetic mice (DB). The KYNA levels in the serum and retina were not significantly different but the AA levels were higher in diabetic mice than in non-diabetic mice. The graphs represent the means ± SD of number of measurements mentioned above. ns = not significant and * *p* < 0.05.

We induced diabetes in mice through i.p. injection of STZ and measured FBG levels. The levels of FBG is higher in STZ-injected mice (231 mg/dL) compared to age matched non-diabetic mice (109 mg/dl). We also determined levels of KYNA in serum and retina of non-diabetic (ND) and diabetic (DB) mice. While there was no significant difference in the KYNA levels between ND and DB mice, a significant increase in AA levels was observed both in the serum and retina of DB mice (Figure 6).

**Figure 7:**
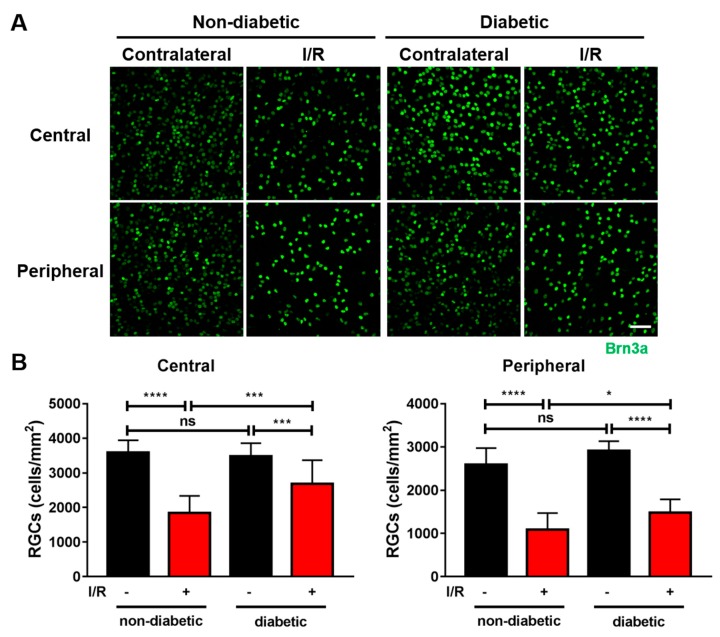
Hyperglycemia inhibited RGC loss in mice subjected to I/R injury. I/R injury was performed in diabetic mice one-week after induction of diabetes. Retinas were dissected out 14 days after I/R injury, flat mounted and immunostained for Brn3a. (*n* = 4), ns = not significant, * *p* < 0.05, *** *p* < 0.001, and **** *p* < 0.0001. Scale bar = 50 μm.

Finally, we tested whether elevation of blood glucose has any effect on RGC survival against I/R injury in diabetic mice. In non-diabetic mice, I/R injury resulted in a significant 49% (*p* < 0.0001) and 58% (*p* < 0.0001) decrease in the Brn3a-positive RGCs in the central and peripheral retina when compared with untreated contralateral eyes (Figure 7). Short-term diabetes did not affect RGC number either in central or peripheral retina. In diabetic mice subjected to I/R injury, the number of Brn3a-positive RGCs decreased by 25% and 42% in the central (*p* < 0.001) and peripheral (*p* < 0.0001) retina when compared with uninjured contralateral eyes. In fact, short-term diabetes resulted in 24% (*p* < 0.001) and 16% (*p* < 0.05) more remaining RGCs in the central and peripheral retina when compared with non-diabetic mice subjected to I/R injury.

## 3. Discussion

The objective of this study was to determine the effects of KYNA on RGCs in the retina. The idea stemmed from previous observations that KYNA is neuroprotective and 3OHKYN is neurotoxic in the brain [33,34,35]. Whether these metabolites in the KP affect RGCs was less studied. During our study, a study by Harper et al. [25] showed that systemic inhibition of KMO protects RGCs in an experimental mouse model for traumatic brain injury, supporting the idea that KP metabolites play a role in RGC health. We found that the absence of KMO led to higher levels of KYN, KYNA and AA in the serum and retina, coincident with a protection against retinal I/R injury. There is evidence for accumulation of extracellular glutamate during ischemic injury to the retina [36], which can activate NMDA receptors and cause RGC death. Further, administration of NMDA receptor antagonists protects RGC death against ischemic injury [37,38]. Therefore, the neuroprotective property of KYNA could be due to its antagonistic activity toward the NMDA receptor [15], and/or anti-inflammatory and antioxidant effects [39,40]. Previous studies have shown that KYNA crosses the blood brain barrier poorly [29,30]. It was therefore reasonable to assume similar poor crossing through the blood retinal barrier. However, our study showed elevated levels of KYNA in the retina upon intravenous administration of KYNA in mice, suggesting some permeability of KYNA across the blood retinal barrier (BRB). We cannot however rule out the possibility that the higher KYNA levels were due to elevated levels in the retinal blood vessels. However, it is remarkable that intravenously administered KYNA protected RGCs from I/R injury, possibly because due to its delivery to RGCs or secondary indirect effects in the retina. Several studies have reported that I/R injury leads to a breakdown of BRB, which results in increased vascular permeability [41,42]. This could be a reason for additional KYNA permeability in I/R injured retinas.

RGCs comprise of multiple subtypes [43]. The number of Brn3a-positive RGCs, but not RPBMS-positive RGCs, was significantly lower in the naïve retinas from KMO KO mice when compared to naïve retinas from WT mice. Our results suggest that in the absence of KMO, an RGC subtype that is positive for Brn3a was reduced possibly due to death or developmental defects. The elevated levels of AA in the retina may have had downstream effects on RGCs. Interestingly, despite its link to several diseases [44], the known in vivo functionality of AA and its derivatives is limited. Of note, despite the absence of KMO, AA could generate neurotoxic 3OHAA [45,46]. Subtle changes in the levels of 3OHAA, a metabolite we were not able to detect, may have had negative effects on RGCs in the naïve retinas from KMO KO mice.

Oxenkrug reported higher levels of KYNA along with other metabolites from the KP in type 2 diabetes patients [47]. It has been reported that a peripheral KMO deficiency leads to metabolic syndrome in schizophrenic patients [48]. Increased metabolism through the KP could lead to a deficiency in pyridoxal 5-phosphate, which is needed by enzymes that metabolize 3OHKYN; this could lead to XA accumulation, which is suggested to cause insulin resistance [49]. It is known that diabetes is a risk factor for glaucoma [50]. FBG levels were higher in KMO KO mice relative to WT mice, suggesting that the mice had developed mild diabetes or that they were in a prediabetic state. Whether KYNA or other metabolites from the KP had a role in elevating FBG in KMO KO mice needs to be verified. None of the co-morbid pathologies of diabetes (reduced body weight, reduced glucose tolerance, and reduced insulin sensitivity) were present in the KMO KO mice. These additional measures do not provide a clear explanation for the elevation in FBG, nor whether this adjustment in glucose homeostasis contributed to the lower number of Brn3a-positive RGCs in KMO KO mice.

Our results indicating that RGCs are better protected from the I/R injury in short-term diabetic mice than in non-diabetic mice agree well with a previous report that showed protection of RGCs during short-term diabetes in experimental glaucoma in rats [51]. Availability of higher levels of glucose for stressed RGCs has been proposed as a mechanism. However, long-term diabetes is known to cause RGC loss in experimental animals [52]. It should be noted, however, that the absence of KMO resulted in only mild elevation of blood glucose (193 ± 10 in KMO KO and 231 ± 36 in STZ-induced diabetes in 2 weeks). Whether the protection was entirely due to higher levels of glucose or elevated KYNA or the combination of the two needs to be investigated further.

Furthermore, a word of caution: systemically elevated KYNA levels in rodents have been shown to generate cognitive deficits similar to those seen in schizophrenia [53]. In contrast, in another study, long-term administration of KYNA in rodents did not cause any detrimental effects [54]. KYNA has been shown to target alpha7 nicotinic acetylcholine receptors (α7nAChRs) in the brain and reduces glutamate and dopamine levels [55,56], and the α7nAChR agonist PNU-282987 has previously been shown to have neuroprotective effects against RGC loss [57]. If KYNA targets α7nAChR in the retina, it may be counterproductive with regards to RGC protection. Thus, increasing KYNA levels locally in the eye might be a useful strategy to prevent RGC death in glaucoma.

In summary, our study showed that the absence of KMO protected RGCs from I/R-induced damage. We propose that this could be due to higher levels of KYNA. The localized delivery of KYNA into the retina may obviate potential systemic effects. Current treatments for glaucoma are primarily through topical eye drops with drugs. Whether such topical delivery of KYNA is a better approach than intravitreal administration needs to be investigated. Thus, further studies are warranted to test the pharmacokinetics and feasibility of using KYNA against RGC death in glaucoma.

## 4. Materials and Methods

All chemicals used were at least of analytical grade. All animal experiments were reviewed and approved by the University of Colorado Denver Institutional Animal Care and Use Committee (IACUC) and performed under adherence to the ARVO Statement for the Use of Animals in Ophthalmic and Vision Research. WT C57BL/6J mice (Stock No: 000664) were obtained from Jackson Laboratories (Bar Harbor, ME, USA). Kmo^tm1a(KOMP)Wtsi^ mice were obtained from the KOMP repository (University of California, Davis, CA, USA).

### 4.1. Administration of KYNA

To check the permeability of exogenously administered KYNA into the retina, KYNA was injected intravenously (via the tail vein) into WT mice at a concentration of 2.5 mg/animal (25 mg/mL stock dissolved in 0.1 N NaOH, diluted in 0.1 M sodium phosphate buffer, and adjusted to pH 7). After 2 h, KYNA levels in the serum and retina were measured as described below. Control animals received 0.1 M sodium phosphate buffer (pH 7) alone.

KYNA was injected intravitreally into WT mice at a concentration of 5 and 10 μg/animal (5 mg/mL stock of KYNA was dissolved in 0.1 N NaOH and diluted in 0.1 M sodium phosphate buffer adjusted to pH 7) immediately after and 24 h after I/R injury. Control animals were intravitreally injected with buffer alone. Intravitreal injection was performed using a 33-gauge needle attached to a Hamilton syringe (Hamilton Bonaduz AG, Bonaduz, Switzerland). The eye lids were carefully parted, and the 33-gauge needle was inserted at a 45° angle into the vitreous, just behind the limbus. One or two microliters of solution was injected in 1 µL increments with a 30 sec gap between each injection. After injection, the needle was slowly withdrawn, and the injected area was treated with a topical antibiotic. Mice were anesthetized with a cocktail containing ketamine and xylazine prior to the intravitreal injections.

### 4.2. Retinal Ischemia Reperfusion Injury (I/R)

Twelve-week-old WT (C57BL/6J) or KMO KO (male) mice were anesthetized with an intraperitoneal injection of ketamine/xylazine confirmed by the toe-pinch pain test. The animals were then placed on a heating pad throughout the procedure to maintain their body temperature. The right eye of each animal was cannulated into the anterior chamber with a 33-gauge needle connected to an elevated saline reservoir. The height of the reservoir was adjusted to achieve an intraocular pressure of 120 mmHg. After 60 min of this procedure, the needle was removed.

### 4.3. Whole Mount Immunostaining of Mouse Retinal Ganglion Cells

The animals were euthanized on day 14 post-I/R injury. The retinas were dissected out and fixed in 4% paraformaldehyde (Cat# 15710, Electron Microscopy Sciences, Hatfield, PA, USA) overnight. The next day, the fixative solution was aspirated, and the retinas were rinsed and permeabilized with PBS containing 0.1% sodium citrate and 0.2% Triton-X100 for 5 min. The retinas were then treated with 5% donkey serum (Cat# 017-000-121, Jackson Immunoresearch labs Inc. West Grove, PA, USA) overnight at 4 °C. Next, the retinas were incubated with Brn3a (C-20) antibody (1:500 dilution, Cat# sc-31984, Santa Cruz Biotechnology, Dallas, TX, USA) and/or with a RBPMS antibody (1:200 dilution, Cat# GTX118619, GeneTex, Irvine, CA, USA) for 3 days at 4 °C. After PBS washes, the retinas were incubated with Alexa Fluor 488-conjugated donkey anti-goat IgG (Cat# A11055) for single staining of Brn3a or donkey anti-goat IgG Texas Red (Cat# PA 1-28662) for Brn3a and donkey anti-rabbit IgG-conjugated Alexa 488 (Cat# A21206) for RBPMS (all 1:1000 dilution, Life Technologies, Carlsbad, CA, USA) for 1 day at 4°C. Using a sharp scalpel blade, the retinas were cut into 4 petal shapes for the flat mounts. The RGC numbers in the confocal microscopic images (Nikon Eclipse Ti, Nikon instruments Inc. Tokyo, Japan) were counted (cells/mm2) in the central and peripheral regions from four quadrants of the whole-mounted retina using the ImageJ software (NIH) for Brn3a or RBPMS in a masked fashion.

### 4.4. Generation of KMO KO Mice Without an Rd8 Mutation

To screen for KMO KO, genomic DNA was isolated from animal tissue using the DNeasy blood and tissue kit (Qiagen, Germany) and used for genotyping, using the CSD-Kmo-F (5′-TTCTGACCCCATCTGTGTCTGTTCC-3′) and CSD-Kmo-ttR primers (5′-ATCAGAGCTCCCTAAATATGGTGGC-3′) for the wild type *Kmo* gene, 649 bp amplicon. The CSD-neoF primer (5′-GGGATCTCATGCTGGAGTTCTTCG-3′) in conjunction with the Kmo-ttR primer were used to confirm the presence of the L1L2_Bact_P cassette; the resulting gene knockout was represented by a 554 bp amplicon.

As Kmo^tm1a(KOMP)Wtsi^ mice were generated on a *C57BL/6N* background, the mice were backcrossed with *C57BL/6J* and screened for the *Rd8* mutation in the *Crb1* gene, which is known to induce retinal degeneration [58]. To generate *Kmo^-/-^ Rd8^WT/WT^* mice, homologous recombination had to occur as both genes are on chromosome 1. The primers used with the isolated genomic DNA to confirm the presence or absence of the *Rd8* mutation were as described by Mehalow et al. [59]. mCrb1-mF1 (5′-GTGAAGACAGCTACAGTTCTGATC-3′), mCrb1-mF2 (5′-GCCCCTGTTTGCATGGAGGAAACTTGGAAGACAGCTACAGTTCTTCTG-3′) and mCrb1-mR (5′-GCCCCATTTGCACACTGATGAC-3′) resulting in the 220 bp WT allele amplicon and/or the 244-bp *Rd8* mutant allele amplicon. Kmo^tm1a(KOMP)Wtsi^ mice were backcrossed with *C57BL/6J* mice and their progeny were bred to establish the KMO KO genotype, which were homozygous for the L1L2_Bact_P cassette, did not contain the *Rd8* mutation and are referred to as KMO KO mice throughout the paper. The absence of the full length KMO WT transcription product was confirmed by RT-PCR. Furthermore, we established the lack of KMO using an activity assay in which the amount of KYN converted to 3OHKYN by a tissue extract was measured via LC-MS/MS, as described below.

### 4.5. Confirmation of the Lack of KMO in the KO Mice

Approximately 20–25 mg each of liver, kidney and retina from WT and KMO KO animals were homogenized in 500 μL Qiazol (Qiagen), and then 100 μL chloroform (Fisher Scientific, Hampton, NH, USA) was added. After vigorous vortexing and centrifugation at 16,100 g for 15 min at 4 °C, RNA was isolated from the aqueous fraction using the RNeasy Micro Kit (Qiagen). RNA was reverse transcribed into cDNA using the QuantiTect Reverse Transcription Kit (Qiagen), followed by RT-PCR. The mouse *Kmo* sense (5′-TTCCAAAGGTGTGCCCATGA-3′) and mouse *Kmo* anti-sense (5′-AAAGTGCACCTTCGCATTGG-3′) primers, which spanned the cassette insertion site in the KO animals, were used for RT-PCR screening. The absence or presence of a 169 bp amplicon after RT-PCR indicated the knockout or expression of WT *Kmo*. GAPDH (primers Cat# Mm.PT.39a.1, IDT, San Jose, CA, USA) or β-actin (sense: 5′- AGAAAATCTGGCACCACACC-3′, anti-sense: 5′- GGGGTGTTGAAGGTCTCAAA-3′) was used as a housekeeping gene.

### 4.6. Measurement of KMO Enzyme Activity in the Liver

We determined KMO enzyme activity in a subcellular fraction from liver extracts from WT and KMO KO mice based on the protocol described by Winkler et al. [60]. The enzyme activity was measured by quantifying 3OHKYN using a Acquity UPLC system (Waters, Milford, MA, USA) in conjunction with MS analysis on a Sciex 4500 Qtrap (Redwood City, CA, USA). Recombinant KMO (Cat# 8050-KM-025, R&D systems Minneapolis, MN, USA) was used as a positive control and the addition of the KMO inhibitor Ro 61-8048 (Tocris, Minneapolis, MN, USA) was used as a negative control. Briefly, the tissue was homogenized in ice cold homogenization buffer (20 mM HEPES pH 7.3 with 0.32 M sucrose, 10 mM KCl, and 1 mM EDTA) and centrifuged at 1000× *g* for 5 min at 4 °C. The obtained pellet was washed four times in homogenization buffer via centrifugation. The pellet was resuspended in homogenization buffer at 2.5 mL/g wet weight original tissue, after which the final protein concentration was adjusted to 1.5 mg/mL. The assay buffer contained a final concentration of 100 mM potassium phosphate (pH 7.4), 200 µM NADPH, 0.4 units/mL glucose 6-phosphate dehydrogenase, and 3 mM D-glucose 6-phosphate. The reaction mixture contained 0.1 mg protein/mL of recombinant KMO or 0.3 mg protein/mL from liver isolate and 200 µM final concentration of L-kynurenine sulfate substrate (Sigma-Aldrich, St. Louis, MO, USA) in a final volume of 40 µL assay buffer. The reaction mixture was incubated for 60 min at 25 °C and the enzyme activity was terminated by the addition of 40 µL of 10% trichloroacetic acid (TCA). The mixture was vortexed and centrifuged (5 min at 10,000× *g* and 4 °C), after which 25 µL of the supernatant was diluted 1:1 with acetonitrile and submitted to LC-MS/MS analysis. The samples were analyzed using an ACQUITY UPLC BEH Amide Column, 130 Å, 1.7 µm, 2.1 × 100 mm column connected to a guard column (Waters) maintained at 40 °C with 1% formic acid (FA) in water (solvent A) and 1% FA in acetonitrile (solvent B) as mobile phases. Chromatography was performed at a flow of 0.5 mL/min. The percentage of solvent A over time was as follows: initial-0.25 min, 5%; 0.75 min, 20%; 1.60 min, 50%; 1.80–3.5 min, 70%; and 3.60 min, 5%. KYN and 3OHKYN (fresh solution) standards were used. The multiple reaction monitoring (MRM) transitions for 3OHKYN and KYN were observed as indicated in Table 1. The product ion 1 MRM transition was used for quantitation (quantifier (QN)), whereas the product ion 2 and 3 MRM transitions were used for confirmation (qualifiers (QL1,2)). The MS/MS conditions were as follows: curtain gas (CUR), 45 mL/min; ion spray voltage (IS), 4.5 kV; temperature, 550 °C; nebulizer gas (GS1), 60 mL/min; heater gas (GS2), 70 mL/min; collisionally activated dissociation (CAD) gas, 2 mL/min; and entrance potential (EP), 10 V.

### 4.7. Measurement of KP Metabolites in the Serum and Retina

Serum and retina samples were analyzed for the levels of KP metabolites using the following procedure. The standards were freshly prepared. Trp and AA were obtained from Sigma-Aldrich; KYN was obtained from Honeywell–Fluka Research Chemicals (Morris Plains, NJ, USA); and KYNA was from Tocris Bioscience (Avonmouth, Bristol, UK). Stock solutions were prepared in 0.1 M formic acid at 10 μM concentration and serially diluted to generate a calibration curve.

To measure the serum KP metabolites, whole blood was collected and incubated at room temperature for 30 min prior to centrifugation at 2000× *g* for 10 min to separate the serum. Next, 47.5 μL serum was adjusted to 50 μL with water followed by the addition of 125 μL of 5 mM ammonium formate containing 0.1% trifluoroacetic acid (TFA). Fifty microliters of ice cold 5% TCA was added to the mixture and mixed well. The samples were centrifuged at 10000× *g* for 10 min and the supernatant was used to measure the KP metabolites. For analysis of KP metabolites in the retina, each retina was homogenized in 50 μL of ice cold 5% TCA. Then, 125 μL of 5 mM ammonium formate containing 0.1% TFA was added to this homogenate and the samples were centrifuged at 10,000× *g* for 10 min. The supernatant was immediately analyzed by LC-MS/MS. Experiments were performed on the abovementioned Waters UPLC—AB Sciex 4500 Qtrap mass spectrometer system using a Acquity HSS T3 1.8 μM 2.1 × 100 mm column with guard column (Waters). The following eluents were used to form a UPLC gradient: MS grade water with 0.12% heptafluorobutyric acid (HFBA) (Sigma-Aldrich) (solvent A) and 80% MS grade acetonitrile in water with 0.12% HFBA (solvent B). The gradient was used for the analysis of the Trp metabolites at 0.6 mL/min with a column temperature of 40 °C. The column was run using the following gradient of solvent A: 98% A from 0–2.2 min, 92% A 2.2–3.3 min, 87% A from 3.3–5 min, 80% A from 5–7.6 min, 66% A from 7.6–7.8 min and 0% A from 8–9.7 min, after which the solvent ratio was returned to 98% A from 10–12 min for re-equilibration. The column flow rate was kept constant at 0.6 mL/min. The analytes were detected using a scheduled MRM. The MS ion source conditions during the experiment were as follows: CUR, 35 mL/min; IS, 2.5 kV; temperature, 650 °C; GS1, 65 mL/min; GS2, 70 mL/min; and EP,10.00 V. The MRM transitions for observed metabolites are indicated in Table 1. For the LC-MS/MS analysis in Figure 6, retinas were weighed, and the KP metabolites were extracted in ice cold methanol: acetonitrile: water (5:3:2) (1 mg retina/0.06 mL), and resuspended in 0.1% formic acid as described before [61]. Serum samples were thawed on ice, then processed in the same manner using 20 μL of serum and 480 μL of the extraction solvent. Samples were analyzed on a 5 min C18 gradient method using a Thermo Vanquish UHPLC coupled to a Thermo Q Exactive mass spectrometer in positive and negative ion modes (separate runs) as described before [62].

### 4.8. Assessment of Capillary Degeneration in the Retina

Capillary bed preparation of WT and KMO KO retinas and quantification of capillary degeneration were performed as previously described [63,64].

### 4.9. Measurement of Serum Insulin Levels by ELISA

Serum was separated as mentioned above and 20 μL was used for the ELISA. Insulin was quantified using a Mouse Insulin ELISA kit (ALPCO, Salem, NH, Cat#80-INSMS-E01) as per manufactures instructions.

### 4.10. Measurement of FBG and ITT

For measurement of FBG, mice were fasted for 6 h and blood glucose levels were determined with tail vein blood using either an ARKRAY GLUCOCARD 01 blood glucose meter or a Contour glucometer (Parsippany, NJ, USA). For ITT, animals were fasted for 6 h, neutral protamine Hagedorn insulin (Cat# HI-313, Eli Lilly, Indianapolis, IN, USA) was administered i.p. (1 unit/kg bw), and blood glucose was measured with tail vein blood drawn at 0, 15, 30, 45, 60, 120 min post insulin injection.

### 4.11. Induction of Diabetes in Mice

Male *C57BL/6J* mice at 3-months of age were made diabetic by i.p. administration of streptozotocin (STZ, MP Biomedicals, Solon, OH, USA), as previously described [64]. Diabetes was confirmed by the elevated fasting blood glucose after 1 week of STZ injection. FBG was measured as described above using ARKRAY GLUCOCARD 01 blood glucose meter (ARKRAY USA, Minneapolis, MN, USA).

### 4.12. Statistics

We used the GraphPad Prism software (Version 7, GraphPad Prism Software, Inc., San Diego, CA, USA) to analyze the data using unpaired t-tests and one-way ANOVA Tukey’s multiple comparisons test. The results are presented as the mean ± SD of the specific number of experiments indicated in the figure legends. The differences were considered significant at *p* < 0.05.

## Figures and Tables

**Table 1:** The multiple reaction monitoring (MRM) transitions for observed metabolites.

**KMO Activity Assay**											
		QN	QL1	QL2		QN	QL1	QL2	QN	QL1	QL2
	Q1	Q3	Q3	Q3	DP	CE	CE	CE	CXP	CXP	CXP
	M/Z	M/Z	M/Z	M/Z	V	V	V	V	V	V	V
KYN	208.90	93.90	145.90	136.00	35	18.80	29.00	16.70	8.40	9.60	7.00
3OHKYN	225.10	110.00	162.00	190.10	30	20.00	20.00	22.00	9.00	11.00	11.00
**Tryptophan Metabolites MRM Transitions**											
		QN	QL1	QL2		QN	QL1	QL2	QN	QL1	QL2
	Q1	Q3	Q3	Q3	DP	CE	CE	CE	CXP	CXP	CXP
	M/Z	M/Z	M/Z	M/Z	V	V	V	V	V	V	V
KYN	209.00	192.00	146.00	94.00	40	12.00	22.50	18.80	10.00	8.00	8.00
Trp	205.00	188.10	146.00	117.90	30	12.80	23.50	33.90	6.80	9.80	8.80
KYNA	190.00	143.90	172.00	162.00	35	25.50	16.00	21.12	12.00	12.20	8.80
3OHKYN	225.20	208.10	110.20	162.20	30	12.93	23.31	28.30	7.97	9.88	5.14
AA	138.10	119.70	92.00	65.10	30	23.16	27.80	37.41	10.19	7.80	10.00
3OHAA	154.00	135.80	108.10	80.10	35	18.50	28.20	34.25	11.04	9.00	7.93

## Supplementary Materials

Supplementary materials can be found at https://www.mdpi.com/1422-0067/21/5/1795/s1.

## Abbreviations

RGC	retinal ganglion cells
I/R	ischemia/reperfusion
KMO	kynurenine 3-monooxygenase
KP	kynurenine pathway
Trp	tryptophan
KYN	kynurenine
KYNA	kynurenic acid
3OHKYN	3-hydroxykynurenine
XA	xanthurenic acid
AA	anthranilic acid
IDO	indoleamine 2,3-dioxygenase
KAT	kynurenine amino transferase
NMDA	N-methyl-D-aspartic acid
FBG	fasting blood glucose
ITT	insulin tolerance test
Brn3a	brain-specific homeobox/POU domain protein 3A
RBPMS	RNA-binding protein with multiple splicing
WT	wild type
KO	knockout
I/R	ischemia/reperfusion
ELISA	enzyme-linked immunosorbent assay
MRM	multiple reaction monitoring
